# Bioinformatics analysis for the identification of key genes and long non-coding RNAs related to bone metastasis in breast cancer

**DOI:** 10.18632/aging.203211

**Published:** 2021-07-05

**Authors:** Xu Teng, Tianshu Yang, Wei Huang, Weishi Li, Lin Zhou, Zihang Wang, Yajuan Feng, Jingyao Zhang, Xin Yin, Pei Wang, Gen Li, Hefeng Yu, Zhongqiang Chen, Dongwei Fan

**Affiliations:** 1 Beijing Key Laboratory for Cancer Invasion and Metastasis Research, Department of Biochemistry and Molecular Biology, School of Basic Medical Sciences, Capital Medical University, Beijing 100069, P.R. China; 2 Department of Orthopaedics, Peking University Third Hospital, Beijing 100191, P.R. China; 3 School of Information Science and Technology, University of Science and Technology of China, Hefei 230026, Anhui, P.R. China; 4 State Key Laboratory of Molecular Oncology, National Cancer Center/National Clinical Research Center for Cancer/Cancer Hospital, Chinese Academy of Medical Sciences and Peking Union Medical College, Beijing 100021, P.R. China

**Keywords:** bone metastasis, breast cancer, lncRNA-mRNA network, disease-causing gene modules

## Abstract

The molecular mechanism of bone metastasis in breast cancer is largely unknown. Herein, we aimed to identify the key genes and long non-coding RNAs (lncRNAs) related to the bone metastasis of breast cancer using a bioinformatics approach. We screened differentially expressed genes and lncRNAs between normal breast and breast cancer bone metastasis samples using the GSE66206 dataset from the Gene Expression Omnibus. We also constructed a differentially expressed lncRNA-mRNA interaction network and analyzed the node degrees to identify the driving genes. After finding potential pathogenic modules of breast cancer bone metastasis, we identified breast cancer bone metastasis-related modules and functional enrichment analysis of the genes and lncRNAs in the modules. Based on the above analysis, we constructed a differentially expressed lncRNA-mRNA network related to bone metastasis in breast cancer and identified core driver genes, including *BNIP3* and the lncRNA RP11-317-J19.1. The role of core driver genes and lncRNAs in the network implies their biological functions in regulating bone development and remodeling. Thus, targeting the core driver genes and lncRNAs in the network may be a promising therapeutic strategy to manage bone metastasis.

## INTRODUCTION

Breast cancer, which originates in the epithelial tissue of the breast [1], is one of the most common malignant tumors in women, accounting for 30% of the cancers diagnosed in women. Continuous improvements in radical mastectomy and the application of various targeted drugs have markedly improved the survival rate and quality of life of patients with breast cancer, but tumor recurrence and metastasis are still the main prognostic factors [2]. Bone metastasis is the most common site of distant metastasis. Bone metastasis occurs in 65 to 75% of patients with metastatic and recurrent advanced breast cancer, and it is found in 27 to 50% of patients at initial diagnosis [3]. Bone metastasis often occurs in the thoracolumbar vertebrae, sacrum, and ribs [4]. Breast cancer patients with bone metastasis often experience bone-related events, such as pain in bones, pathological fractures, vertebral compression or deformation, spinal cord compression, and hypercalcemia, which seriously affect their quality of life [5].

Metastasis is the main cause of death in approximately 30% of patients with breast cancer, with a higher incidence of bone metastasis. Although breast cancer is peculiarly prone to bone metastasis, our understanding of the molecular mechanism of bone metastasis in breast cancer is still limited. Induced tumor-suppressing mesenchymal stem cells protect bones from metastasis in breast tumors [6] and the bone microenvironment and soluble factors participate in breast cancer bone metastasis. In this complex signaling network, interleukin is a key regulator, affecting the differentiation and function of osteocytes, as both cancer cells and osteocytes secrete interleukin and express the corresponding receptors [7]. MicroRNAs and long non-coding RNAs (lncRNAs), as non-coding RNAs that regulate the expression of target genes, also participate in bone metastasis [8]. It has been reported that miR-214 promotes osteolytic bone metastasis of breast cancer by targeting TRAF3. By detecting the expression profile of miRNAs produced by osteoclasts in human bone specimens, it was discovered that miR-214-3p is significantly upregulated in breast cancer patients with osteolytic bone metastasis. Moreover, miR-214-3p directly targets *Traf3* mRNA to promote osteoclast activity and bone resorption activity [9]. In addition, the ROR1-HER3-lncRNA axis regulates the Hippo-YAP pathway to promote bone metastasis in breast cancer. NRG1 triggers HER3 phosphorylation, and then recruits the lncRNA MAYA and methylates MST1, activating YAP and its target genes, which eventually leads to the induction of osteoclast differentiation and bone resorption by cancer cells [10]. The lncRNA transcription factor homeobox B13 (HOXB13) has been identified as an upstream regulator of bone metastasis-related signals in prostate cancer. HOXB13 promotes the bone metastasis of metastatic prostate cancer by regulating the production of CCL2/CCR2 cytokines and integrin signals in an autocrine and paracrine manner [11].

This study aimed to identify genes and lncRNAs that are related to bone metastasis in breast cancer to determine the molecular mechanism underlying bone metastases in breast cancer. The key genes and lncRNAs identified herein provide a theoretical foundation for the intrinsic molecular mechanism of breast cancer bone metastasis.

## RESULTS

### Screening of differentially expressed genes

The differentially expressed genes and lncRNAs in 12 breast cancer bone metastasis samples and 12 normal samples were identified using the R limma package for differential expression. We used the breast cancer bone metastasis expression profile in the GSE66206 dataset. A total of 133 differentially expressed genes and 23 differentially expressed lncRNAs (fold change >1.2 or fold change <-6/5) were identified. Among them, 27 genes and 3 lncRNAs were significantly differentially expressed (p < 0.05) (Table 1).

**Table 1:** The Gene ontology annotation of the differentially expressed genes between the breast cancer bone metastasis samples and normal samples (GSE66206 from GEO).

**Gene symbol**	**Gene ID**	**Biological Process (GO)**
CSPP1	79848	GO:0051781 positive regulation of cell division
COQ10B	80219	GO:0006743 ubiquinone metabolic process
FLVCR1	28982	GO:0043249 erythrocyte maturation
IDI1	3422	GO:0050993 dimethylallyl diphosphate metabolic process
PTP4A1	7803	GO:0030335 positive regulation of cell migration
ODC1	4953	GO:0009445 putrescine metabolic process
JMJD1C	221037	GO:0033169 histone H3-K9 demethylation
BZW1	9689	GO:0045296 cadherin binding
IRF1	3659	GO:0034124 regulation of MyD88-dependent toll-like receptor signaling pathway
KDM6B	23135	GO:0071557 histone H3-K27 demethylation
IFRD1	3475	GO:0048671 negative regulation of collateral sprouting
RBM25	58517	GO:0000381 regulation of alternative mRNA splicing, via spliceosome
NCOA4	8031	GO:0006879 cellular iron ion homeostasis
SERPINA1	5265	GO:0048199 vesicle targeting, to, from or within Golgi
HSPB1	3315	GO:0038033 positive regulation of endothelial cell chemotaxis by VEGF-activated vascular endothelial growth factor receptor signaling pathway
BNIP3	664	GO:1902109 negative regulation of mitochondrial membrane permeability involved in apoptotic process
LARP4	113251	GO:0034250 positive regulation of cellular amide metabolic process
UXT	8409	GO:0047497 mitochondrion transport along microtubule
ZBTB10	65986	GO:0046872 metal ion binding
LAMTOR1	55004	GO:0060620 regulation of cholesterol import
MRPL51	51258	GO:0070126 mitochondrial translational termination
ELF2	1998	GO:0050855 regulation of B cell receptor signaling pathway
TPD52	7163	GO:0030183 B cell differentiation
PAQR5	54852	GO:0048477 oogenesis
CYB5D1	124637	GO:0046872 metal ion binding
GABRB2	2561	GO:0004890 GABA-A receptor activity
ABHD2	11057	GO:0042562 hormone binding

### Constructing an interaction network related to bone metastasis in breast cancer

**Figure 1:**
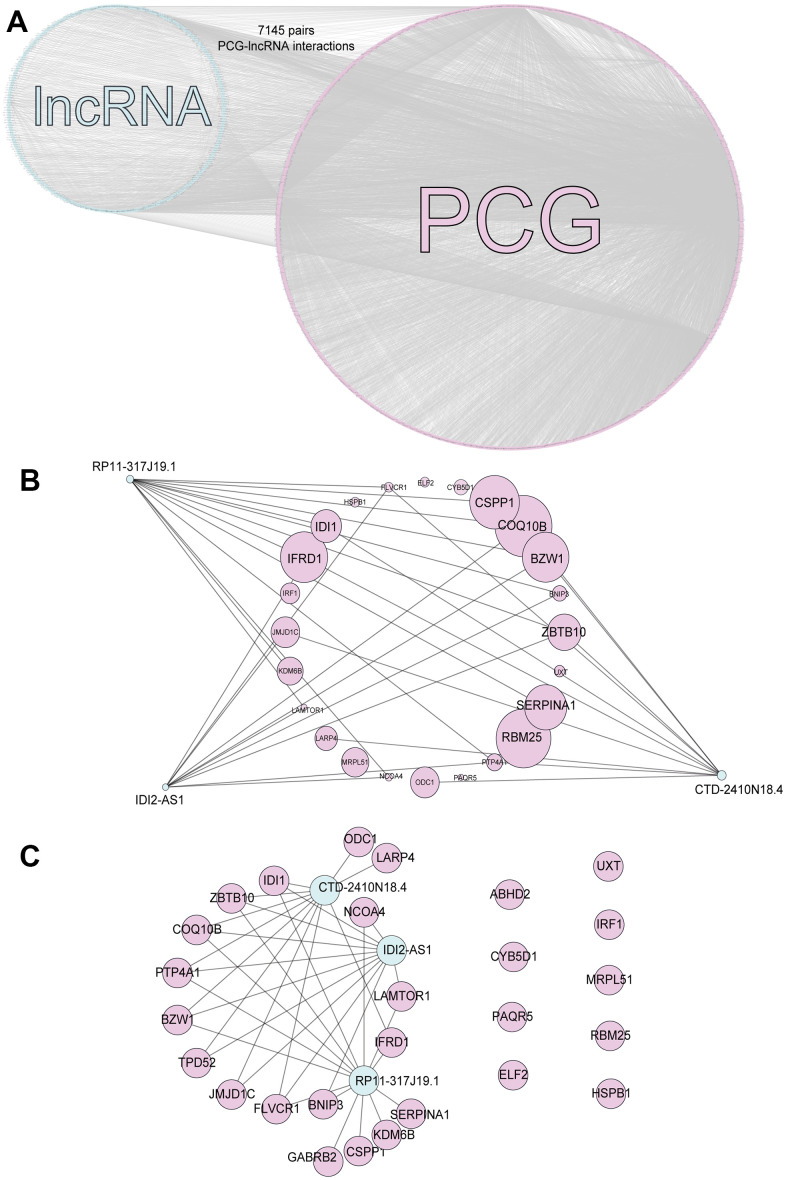
**PCG-lncRNA interactions.** (**A**) Network of differentially expressed PCG-lncRNA interactions. (**B**) Core driver gene network. (**C**) Interactions between differentially expressed genes and lncRNAs.

**Figure 2:**
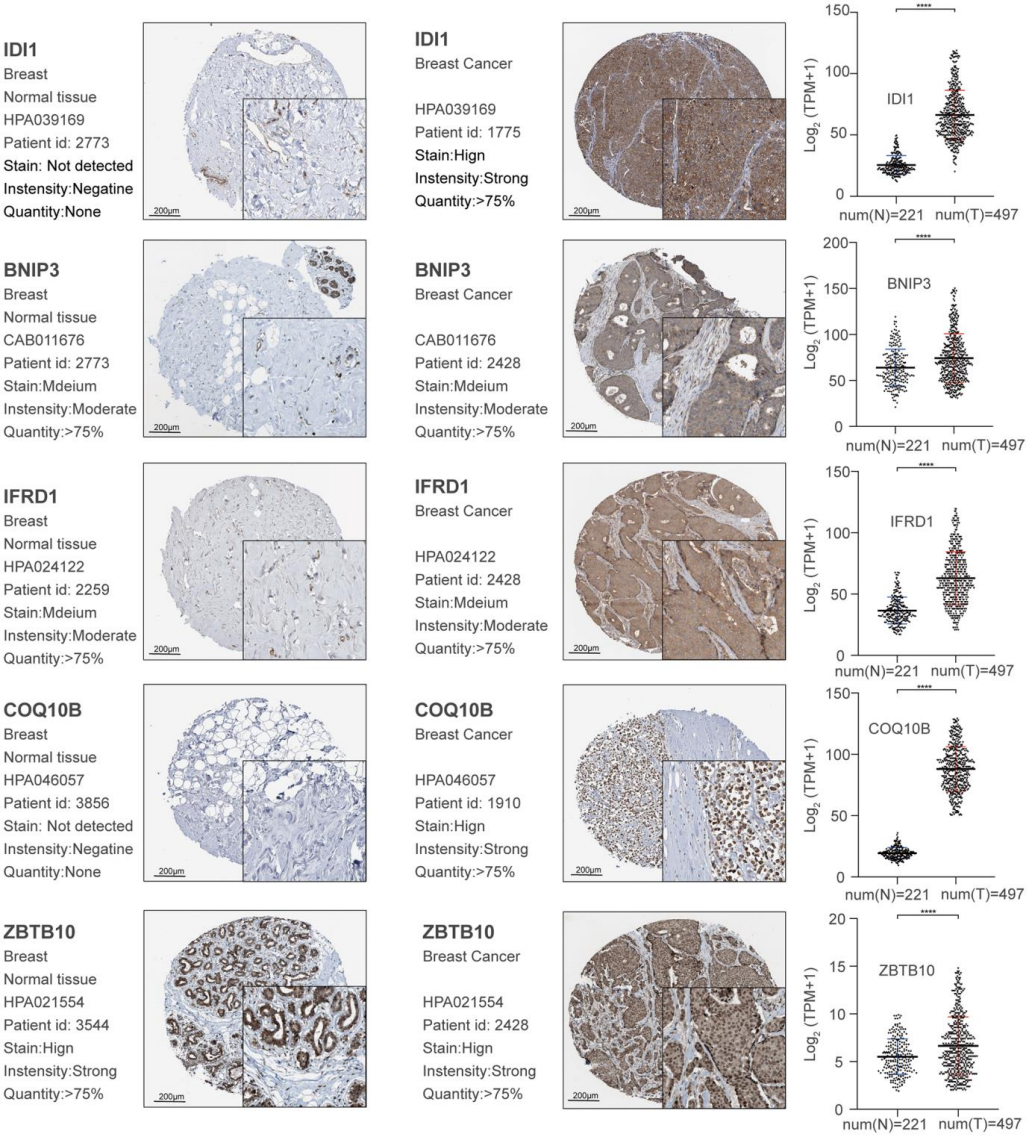
Validation of significant core genes in breast cancer bone metastasis via immunohistochemistry and RNA expression analysis.

We calculated Spearman correlation coefficients for the co-expression of PCGs and lncRNAs, which we corrected using the rank-sum test. We identified 747,870 PCG-lncRNA, PCG-PCG, and lncRNA-lncRNA interactions, 7,451 of which were differentially expressed PCG-lncRNA interactions (| r | ≥ 0.3, p < 0.05). We then visualized the network (Figure 1A) and analyzed the node degrees. Cytoscape analysis revealed that the degree of the network nodes ranged from 1 to 905. To identify the hub nodes in the network, we set the threshold to 20 and extracted all possible core driver genes. A total of 30 different core driver genes were identified (Figure 1B). We also identified regulatory interactions between 27 differentially expressed genes and 3 lncRNAs (RP11-317J19.1, CTD-2410N18.4, IDI2-AS1) (Figure 1C). We consulted the Human Protein Atlas Interactive Analysis to validate the expression of the core driver genes, including *IDI1*, *BNIP3*, *IFRD1*, *COQ10B*, and *ZBTB10* (Figure 2), at the protein and RNA levels in breast cancer. Immunohistochemistry and RNA expression analysis indicated that IDI1, *BNIP3*, *IFRD1*, and *ZBTB10* were significantly differentially expressed in cancer and healthy tissues.

### WGCNA of co-expressed genes

**Figure 3:**
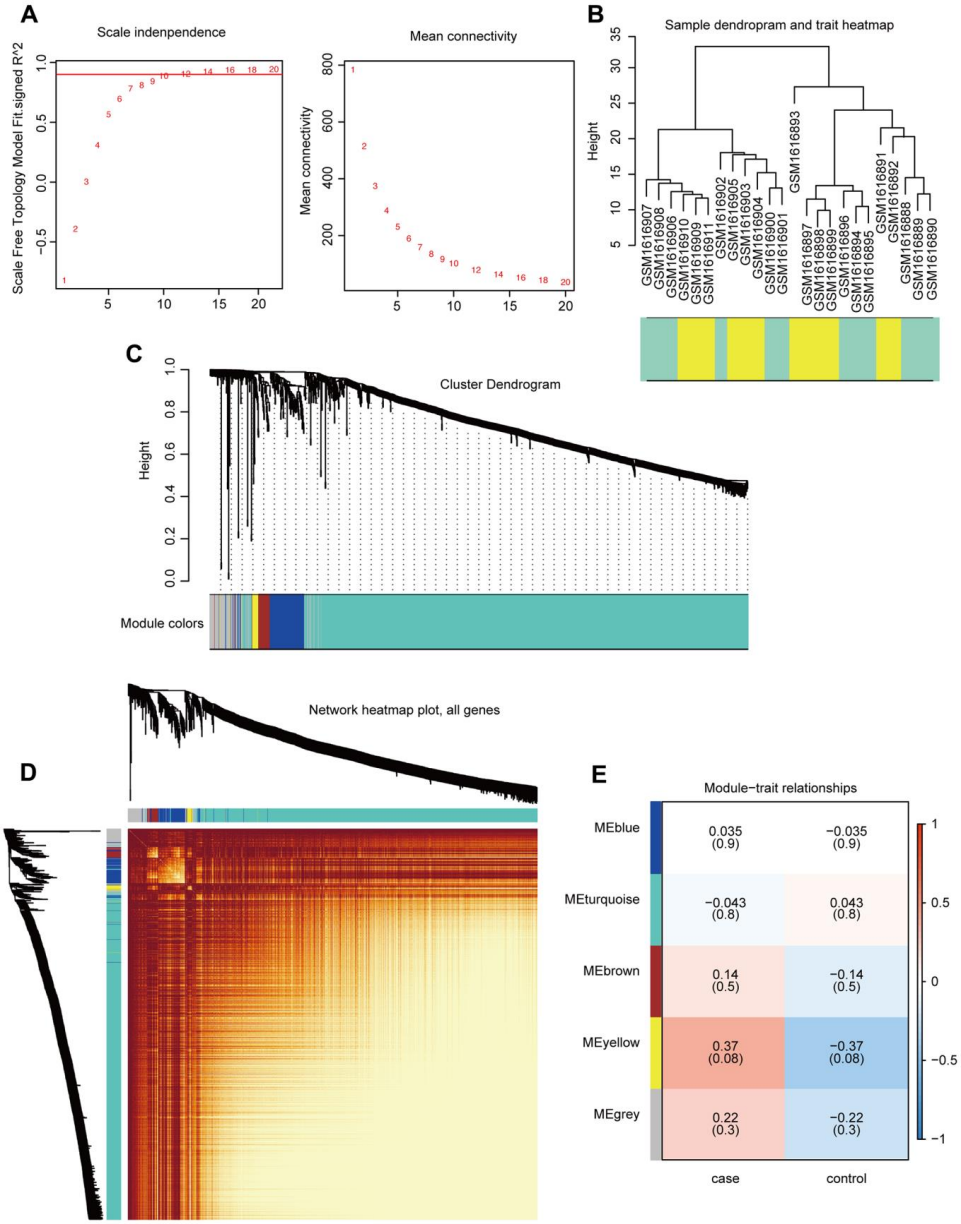
**Construction and analysis of co-expression modules.** (**A**) Optimal threshold selection map for breast cancer bone metastasis co-expression. (**B**) Clustering of breast cancer bone metastasis samples. (**C**) Phylogenetic tree of module clustering. (**D**) Co-expression analysis heat map. (**E**) Breast cancer bone metastasis modules and traits.

Gene expression profiles for 1,533 PCG and lncRNAs related to breast cancer bone metastasis were constructed using the WGCNA R package to build a co-expression network (merge cut height = 0.25, verbose = 3). The optimal threshold for the WGCNA was 12 (Figure 3A). The sample clustering chart is shown in Figure 3B. We excavated five modules from the breast cancer bone metastasis gene expression profile, including blue (112 genes), turquoise (1,274 genes), brown (37 genes), yellow (20 genes), and gray (90 genes) modules (Figure 3C). The results of the co-expression analysis are shown in Figure 3D. Relationships between the various modules and traits related to bone metastasis in breast cancer are shown in Figure 3E. The genes in the yellow module are related to the disease characteristics of breast cancer bone metastases and breast cancer. In addition, the occurrence of bone metastases positively correlated with the expression of the genes in the yellow module.

### Functional enrichment analysis and verification

**Figure 4:**
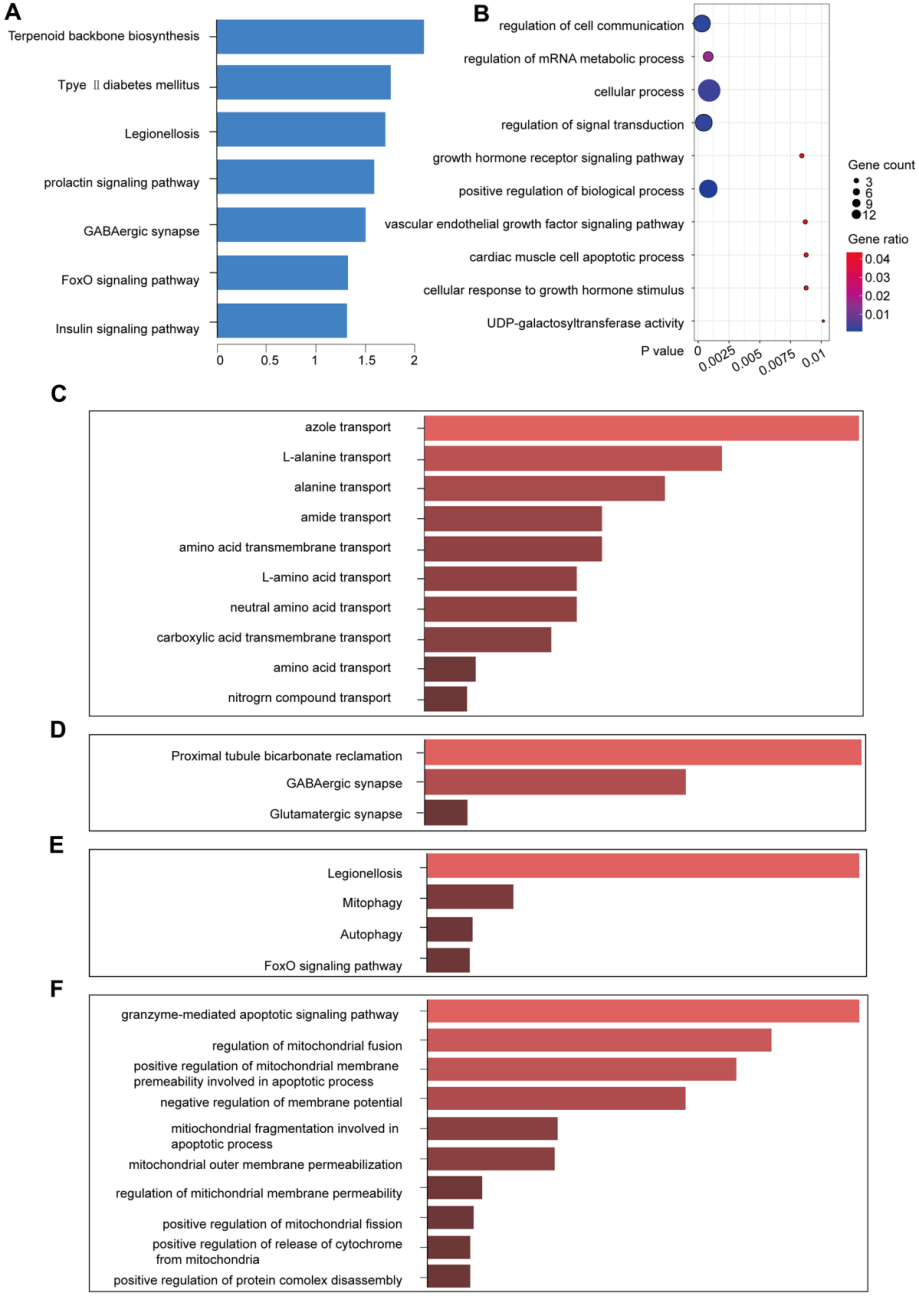
**KEGG and GO function enrichment analyses.** (**A**) KEGG pathway enrichment in the yellow module. (**B**) GO pathway enrichment in the yellow module. (**C**) GO enrichment of lncRNAs in the yellow module. (**D**) KEGG enrichment of the functions of the lncRNAs in the yellow module. (**E**) KEGG pathway enrichment for the interactions of the differentially expressed genes in the yellow module. (**F**) GO pathway enrichment for the interactions of the differentially expressed genes in the yellow module.

The yellow module comprised 14 genes (Table 2). We used KOBAS to perform functional enrichment analysis using GO and KEGG for the genes in the yellow module [12]. With a significance threshold of p < 0.05, we identified seven significant KEGG functional pathways, which revealed that the core genes in the yellow module were enriched in the prolactin signaling pathway [13] (Figure 4A). GO enrichment analysis revealed 300 related GO functions (p < 0.05) involving a large number of pathways that might be related to cancer development, including the regulation of signal transduction (GO: 0009966, p = 0.00017), cellular processes (GO: 0009987, p = 0.00024), cellular communication regulation (GO: 0010646, p = 0.00030), and positive regulation of biological processes (GO: 0048518, p = 0.000826) (Figure 4B).

**Table 2:** The GO annotations of the genes in the yellow module.

**Gene symbol**	**Gene ID**	**Biological process (GO)**
IDI1	3422	GO:0050993 dimethylallyl diphosphate metabolic process
PTP4A1	7803	GO:0030335 positive regulation of cell migration
BNIP3	664	GO:1902109 negative regulation of mitochondrial membrane permeability involved in apoptotic process
NCKAP5	344148	GO:0007019 microtubule depolymerization
SOCS2	8835	GO:0060396 growth hormone receptor signaling pathway
ADAMTS3	9508	GO:1900748 positive regulation of vascular endothelial growth factor signaling pathway
ANXA11	311	GO:0032506 cytokinetic process
DYRK1A	1859	GO:0043518 negative regulation of DNA damage response, signal transduction by p53 class mediator
EPS8L2	64787	GO:1900029 positive regulation of ruffle assembly
DISP1	84976	GO:0007225 patched ligand maturation
WDFY3	23001	GO:0035973 aggrephagy
SLC38A5	92745	GO:1904557 L-alanine transmembrane transport
NOVA1	4857	GO:0120163 negative regulation of cold-induced thermogenesis
SLC38A3	10991	GO:2000487 positive regulation of glutamine transport

Enrichr was used to enrich the KEGG and GO functions of the five lncRNAs in the yellow module (p < 0.05). Some pathways that were enriched for the lncRNAs were related to amino acid transport (Figure 4C, 4D), including alanine transport (GO: 0015808, p = 0.00249) and amino acid transmembrane transport (GO: 0003333, p = 0.00747). We identified the GABAergic synapse (p = 0.02205) in the lncRNA-enriched KEGG pathway, which is closely related to breast cancer metastasis [14, 15]. Therefore, the yellow module was likely to be a pathogenic module.

**Figure 5:**
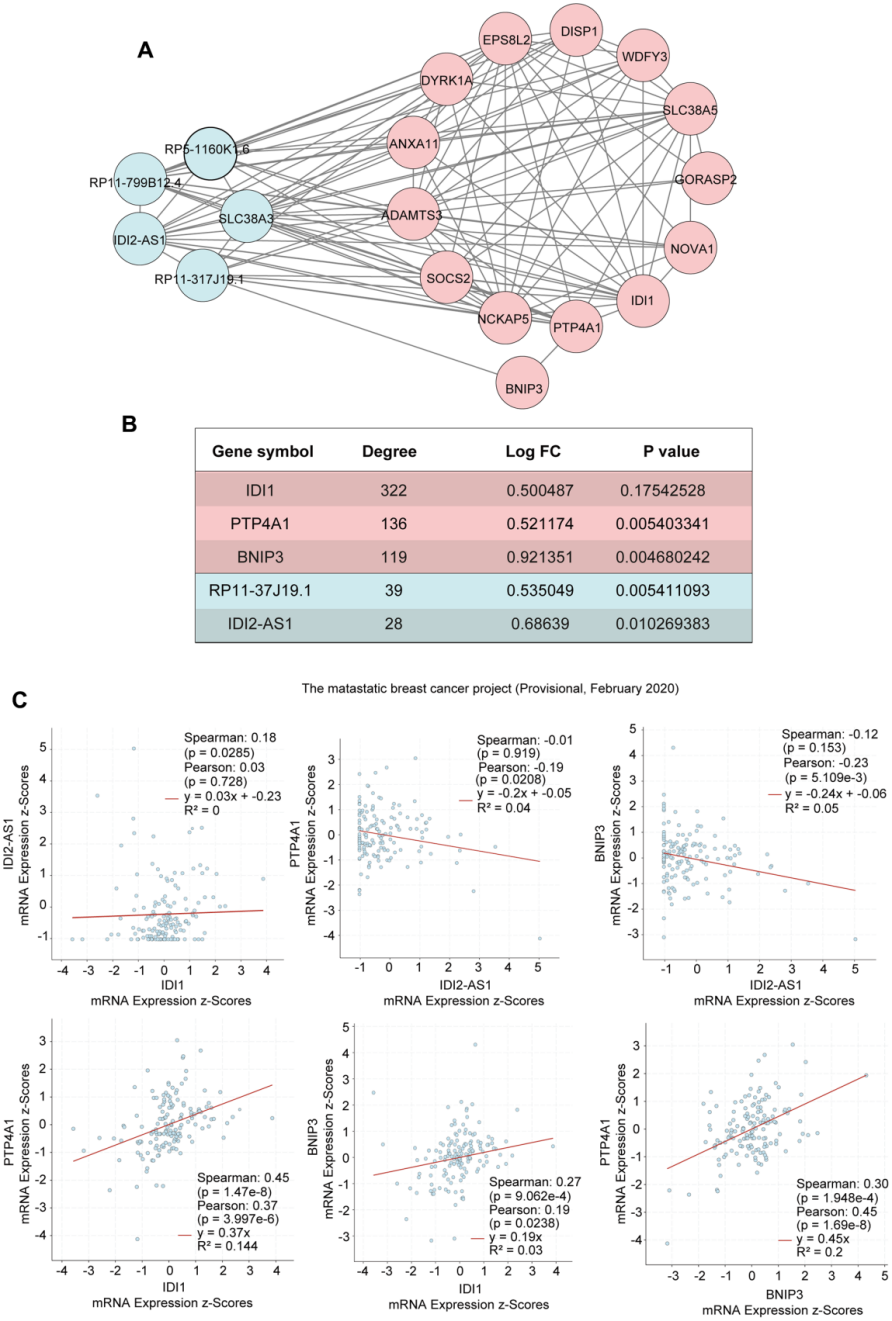
**Interactions of the genes in the yellow module.** (**A**) The interactions between five differentially expressed genes and differentially expressed lncRNAs in the yellow module. (**B**) The five differentially expressed genes in the yellow module. (**C**) Gene correlation scatter plots of the yellow module. The Pearson correlation coefficients of *IDI2*-*AS1* and *IDI1*, *IDI2*-*AS1* and *PTP4A*, *IDI2*-*AS1* and *BNIP3*, *PTP4A* and *IDI1*, *BNIP3* and *IDI1*, and *PTP4A* and *BNIP3*.

**Figure 6:**
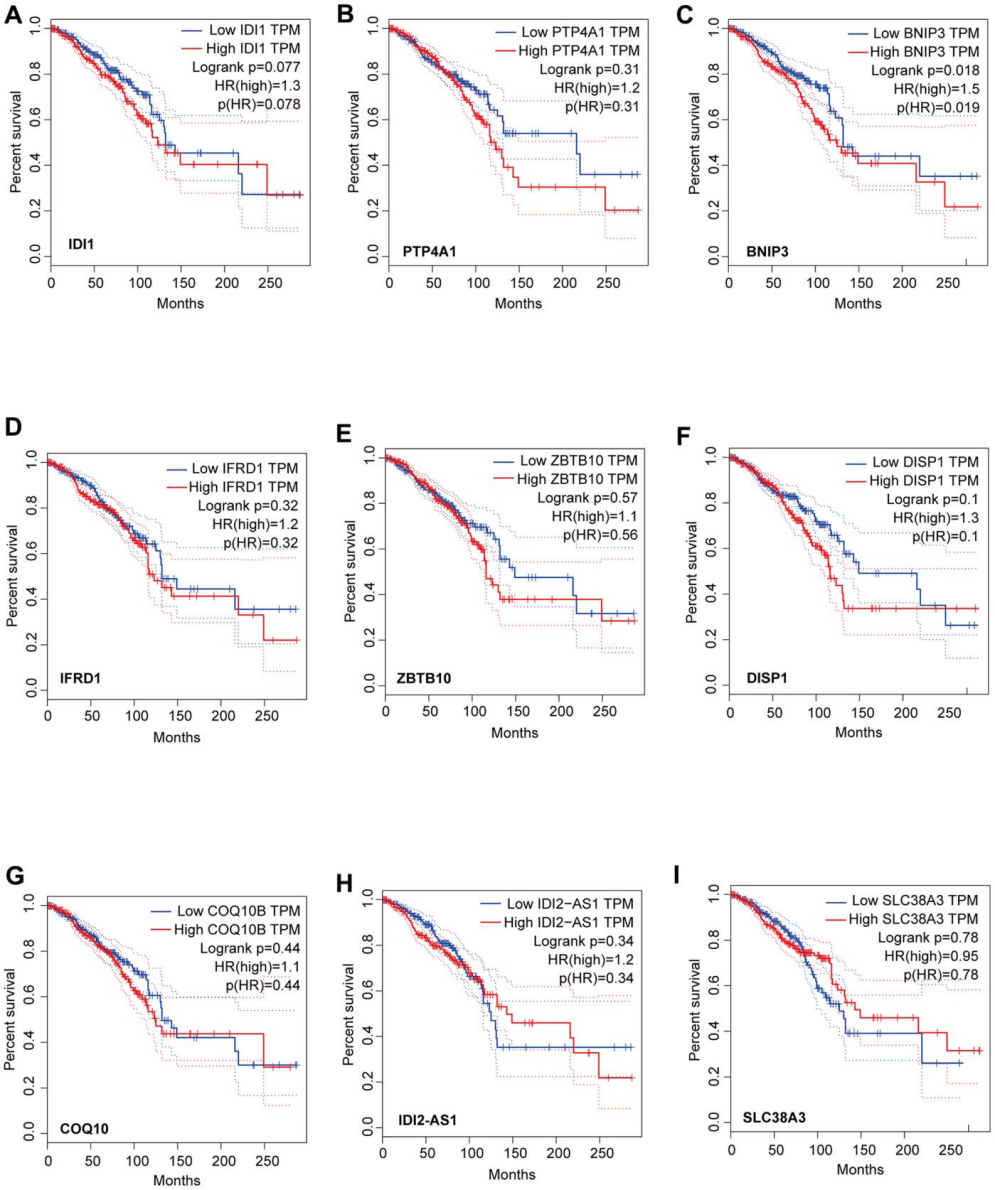
**Analysis of overall survival based on the expression of the seven genes and two lncRNAs in the core driver gene network.** Survival was measured using Kaplan-Meier analysis based on the expression of *IDI1* (**A**), *PTP4A1* (**B**), *BNIP3* (**C**), *IFRD1* (**D**), *ZBTB10* (**E**), *DISP1* (**F**), *COQ10B* (**G**), *IDI2*-*AS1* (**H**), and *SLC38A3* (**I**). The X-axis displays the survival time (Months), and the y-axis displays the percentage survival.

We identified that the lncRNA RP11-317-J19.1 and *PTP4A1* acted on *BNIP3*, which plays a key role in cell apoptosis and autophagy (Figure 4E, 4F) [16–19]. The gene-lncRNA interaction diagram revealed a subtle interaction between the five differentially expressed genes and differentially expressed lncRNAs in the yellow module (Figure 5A, 5B). To validate the gene-lncRNA interactions in the yellow module, we created gene correlation scatter plots for *IDI2*-*AS1* and *IDI1*, *IDI2*-*AS1* and *PTP4A*, *IDI2*-*AS1* and *BNIP3*, *PTP4A* and *IDI1*, *BNIP3* and *IDI1*, as well as *PTP4A* and *BNIP3* (Figure 5C). The results revealed that the expression levels of *BNIP3* and *PTP4A* were significantly positively correlated with that of *IDI1* (p < 0.05), whereas those of *BNIP3* and *PTP4A* were significantly negatively correlated with those of *IDI2*-*AS1* (p < 0.05). Moreover, the Kaplan-–Meier plots showed that seven genes and two lncRNAs in the core driver gene network and co-expression yellow module were correlated with overall survival, including *IDI1*, *PTP4A1*, *BNIP3*, *IFRD1*, *ZBTB10*, *DISP1*, *COQ10B*, *IDI2*-*AS1*, and *SLC38A3* (Figure 6A–6I).

## DISCUSSION

Herein, we identified the genes and lncRNAs that are related to bone metastasis in breast cancer. By comparing the differentially expressed genes identified in our screening with regard to the genes related to breast cancer bone metastasis in the NCBI database, we identified genes with verified links to breast cancer bone metastasis, such as *HSPB1* and *PRL*, in our dataset, thereby validating our approach. *HSPB1* expression is associated with a variety of human cancers with poor clinical prognosis. Furthermore, the *HSPB1*-encoded protein promotes cancer cell proliferation and metastasis, while protecting cancer cells from apoptosis. In addition, prolactin promotes breast cancer bone metastasis [20–22]. The expression of the prolactin receptor modulates the microenvironment to induce osteoclast formation.

Through functional analysis of the lncRNA RP11-317-J19.1, *PTP4A1*, and *BNIP3*, we found that the interaction pairs formed by these genes participate in programmed cell death via apoptosis and autophagy [16–19]. The protein encoded by *PTP4A1* is a cell-signaling molecule with a regulatory role in various processes, such as cell proliferation and migration; therefore, the dysregulation of this protein may also be involved in metastasis [23–27]. *BNIP3* encodes a mitochondrial protein that contains a BH3 domain and functions as a pro-apoptotic factor. *BNIP3* silencing may be mediated by the lncRNA RP11-317-J19.1, allowing the protein encoded by *PTP4A1* to function normally, which may induce the uncontrolled proliferation and migration of breast cancer cells. *PTP4A1* is highly expressed in several cancer types, and the overexpression of *PTP4A1*, which is associated with aggressive tumor characteristics, may be regulated by the PI3K/AKT pathway [28]. *PTP4A1* expression is regulated by microRNAs that control cellular processes in breast cancer, whereas miR-601 targets *PTP4A1* to inhibit breast cancer growth and invasion [24]. The function of BNIP3 is similar to that of PTP4A1, which includes the inhibition of cancer aggression. *PTP4A1* can be modulated by the lncRNAs HULC and RP4 in response to cellular injury [29, 30]. These findings suggest that RP11-317-J19.1, *PTP4A1*, and *BNIP3* may play crucial roles in restraining cancer aggressiveness; thus, they serve as strong predictors of breast cancer bone metastasis.

In conclusion, we constructed a differentially expressed lncRNA-mRNA network related to bone metastases in breast cancer and identified core driver genes. We found that expression modules related to alanine transport and amino acid transmembrane transport were differentially regulated in the bone metastasis and normal samples. Our results reveal key genes and lncRNAs, including *BNIP3* and RP11-317-J19.1, that are related to breast cancer bone metastasis. Our findings lay the foundation for understanding the molecular basis of breast cancer bone metastasis and will be useful for future therapeutic studies.

## MATERIALS AND METHODS

### Gene expression data

We downloaded the gene chip expression data from the GSE66206 dataset and probe annotation files from mouse breast cancer bone metastasis data from the Gene Expression Omnibus (GEO) database (n = 12 normal samples and n = 12 breast cancer bone metastasis samples). The probe annotation files include all probe ID files and probe sequence files for the platform.

### Preparation of the data for probe re-annotation

We downloaded the V19 version of the human protein-coding gene reference transcript sequence and lncRNA reference genomic sequence data from the GENCODE database. The probe sequences of the Affymetrix chip GPL6246 platform used for analyzing the GSE66206 dataset were downloaded from GEO.

### Chip probe re-annotation

First, we used a library comprising the human protein-coding gene (PCG) reference transcript sequence data and lncRNA reference genome sequence data in fasta format from GENCODE to build our database. Then, based on the constructed transcription and lncRNA libraries, we re-annotated the probe sequences of GSE66206 via the blast algorithm in preparation for the construction of mRNA and lncRNA expression profiles. During re-annotation, we ensured that all the remaining probes met the following conditions: 1) The probe sequence fell entirely within the transcript sequence of the PCG or lncRNA and matched exactly; 2) if a probe sequence aligned with the transcripts of multiple PCGs or lncRNAs, it was filtered out; 3) each PCG or lncRNA was supported by at least two probe sequences.

### Differential expression analysis

First, we assigned the appropriate gene symbol to each probe based on the re-annotation and calculated the mean expression for all probes corresponding to the same symbol to determine the expression of the genes in breast cancer bone metastasis and normal samples. The gene expression profiles and lncRNA expression profiles were determined using the R package limma to analyze the differentially expressed genes and differentially expressed lncRNAs between the breast cancer bone metastasis and normal samples. The threshold for differential expression was fold change > 1.2 or fold change < 5/6, where p < 0.05. We compared the screened differentially expressed genes with the related genes on the National Center for Biotechnology Information (NCBI) database to obtain verified or potential genes.

### Construction of an interaction network related to bone metastasis in breast cancer

The Spearman correlation coefficient was calculated from the gene and lncRNA expression data of the breast cancer bone metastasis and normal samples, and a rank-sum test was performed to construct a differentially expressed lncRNA-mRNA interaction network for breast cancer bone metastasis (coefficient | r | ≥ 0.3, p < 0.05 determined by rank-sum test). We visualized the network and analyzed the node degrees to identify the core driving genes.

### Weighted correlation network analysis of co-expressed genes

Co-expression analysis of all genes and lncRNAs associated with breast cancer bone metastasis was performed using the weighted correlation network analysis (WGCNA) R package. We used the WGCNA algorithm to mine the co-expressed gene modules, and then analyzed the associations between these modules and the sample phenotype. The identified breast cancer metastasis-related modules were displayed in the network using Cytoscape.

### Functional enrichment analysis of the genes and lncRNAs in the breast cancer metastasis-related modules

Functions and associated pathways of the breast cancer bone metastasis-related and differentially expressed genes, as well as lncRNAs, were enriched using KOBAS and Enrichr, respectively, via gene ontology (GO) and Kyoto Encyclopedia of Genes and Genomes (KEGG) analyses.
